# Bilateral Proximal Femur Fractures in a Patient with Renal Tubular Acidosis: A Case Report

**DOI:** 10.5704/MOJ.1803.009

**Published:** 2018-03

**Authors:** SS Charl, S Shahrul-Hisham, S Mohamad-Sha

**Affiliations:** Department of Orthopaedics, Hospital Selayang, Selayang, Malaysia; ^*^Department of Orthopaedics, Universiti Teknologi MARA, Sungai Buloh, Malaysia

**Keywords:** pathological fractures, renal tubular acidosis (RTA), bilateral proximal femur fractures, osteomalacia

## Abstract

The diagnosis of pathological fractures is on the rise. The morbidity involved does not only burden the patient and their families but it has a great toll on the healthcare system as well. Early identification of the patient at risk is an invaluable tool to cut cost and improve the patient’s quality of life. Multiple renal pathologies have been highlighted in relation to the risk of pathological fractures; however, complications in renal tubular acidosis have been rarely documented. Nevertheless, prompt action with adequate and relevant patient education ultimately can reduce the associated morbidity. We present a case of poor control of the disease and its debilitating pathological fracture complications.

## Introduction

Renal tubular acidosis (RTA), a rare clinical syndrome with a chronic course, is a disease of the kidney which results in systemic metabolic acidosis^1^. RTA, due to an impaired renal acid excretion, results in distortion of the calcium and phosphate homeostasis leading to enhanced bone resorption^2^. This constellation of events results in osteomalacia which carries the risk of pathological fractures^2^. The fracture incidence has no predilection towards any specific bone or site of bone. Current literature does highlight the increased risk of pathological fracture in renal osteodystrophy, but without much attention to RTA. Despite its risk of fractures, it is uncommon for RTA in an adult patient to present with bilateral proximal femoral fractures. We highlight the clinical importance of identifying this rare group of patients early in an attempt to reduce its associated morbidity and mortality. The pathophysiology and outcome of RTA are discussed to enhance the knowledge of the orthopaedic fraternity for better management for such patients.

## Case Report

A 44-year old female presented to our centre in late 2015 with bilateral lower limb pain and weakness following trivial trauma. She was known to have Type 1 renal tubular acidosis on regular follow-up with the nephrology unit since the age of 35. Prior to the admission, the patient was ambulating mostly independently with an occasional walking aid due to left hip pain. Unfortunately, a trivial fall landing in the sitting position following a vasovagal episode had rendered the patient immobile. On arrival at the emergency unit, she was conscious and alert. Though her vital signs were found to be normal, she appeared lethargic, albeit able to converse and give a full history. Clinically, systemic examination was unremarkable except for tenderness over both hip regions. Her main initial investigations suggested metabolic acidosis with a blood pH of 7.220 and bicarbonate of 14.4mmol/L. Total white blood count was elevated at 17 x 10^3^ mmol/L. Renal profile revealed mild dehydration with a blood urea of 4.7 mmol/L and blood creatinine at 127 mmol/L, which was later corrected during her admission. Her potassium level was normal at 3.9mmol/L. She had hypocalcemia at 1.60mmo/l and hypophosphatemia at 0.59mmol/l. Albumin levels were normal at 35g/dl, but alkaline phosphatase (ALP) was mildly elevated.

All investigations pointed towards poor control of her renal tubular acidosis leading to a systemic metabolic state with hypocalcemia and hypophosphatemia. The raised ALP was likely due to increased osteoblastic activity, a result of the osteomalacia. Further history taking revealed that the patient had not been compliant with the Shohl’s solution (mixture of citric acid and sodium citrate) which was prescribed by the nephrologist, claiming it to be distasteful. Further history revealed a previous fracture neck of left femur in 2010 which had been managed with internal fixation. Poor compliance to her RTA treatment was established after a thorough review. Despite suffering from a chronic renal disorder, the patient had no evidence of renal failure as the glomerular function was normal with no uremia. Therefore, we decided to distance the patient’s case from that of those with chronic renal failure with renal osteodystrophy.

**Fig. 1:**
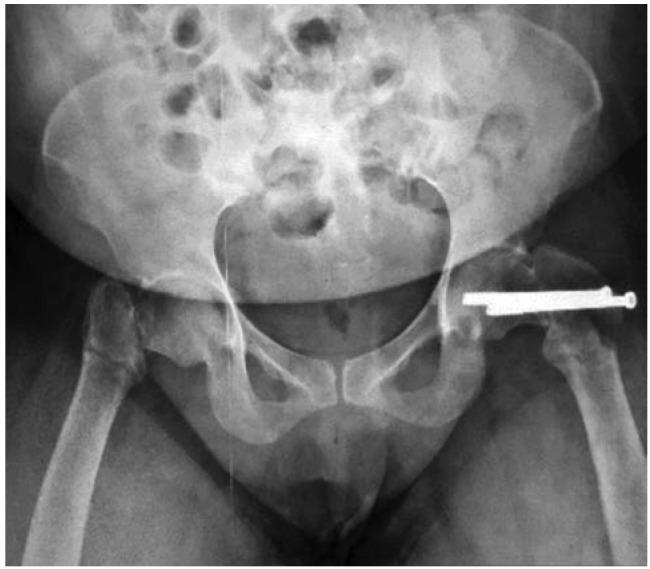
Pelvis AP radiograph at admission showing right sided transcervical neck of femur fracture with left intertrochanteric fracture. Two screws intact in the left neck of femur from previous fixation.

Plain radiographs (Fig. 1) revealed left intertrochanteric fracture with previous screw fixation for fracture of neck of left femur intact, with a contralateral right neck of femur fracture. While awaiting surgery, she was co-managed by a multi-speciality team which included the orthopaedic, nephrology and medical unit. Bilateral lower limb skin traction was applied till the day of surgery.

**Fig. 2:**
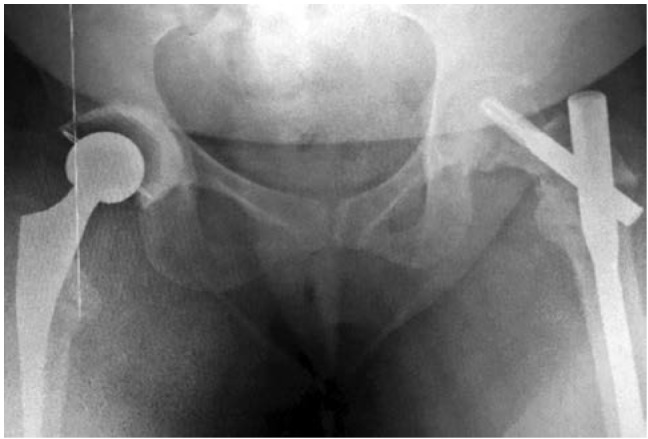
Pelvis AP radiograph showing right total hip arthroplasty and left proximal femoral nail.

The bilateral proximal femoral fractures were treated surgically. A right total hip arthroplasty and left proximal femur nail (Synthes A2) fixation were performed in one setting. We performed a right total hip arthroplasty as the fracture involved an intracapsular component. As for the left sided extracapsular intertroncteric fracture, an intramedullary internal fixation was deemed best in view of the healed previous neck of femur fracture. The decision also was influenced by the need to provide primary stability in either limb to allow early rehabilitation, thus the decision for right total hip arthroplasty. The patient had an uneventful surgery and recovery and was discharged well with regular follow ups in the clinic to monitor progress with plain radiographs (Fig. 2). Awareness of the patient's current condition as well as emphasis over the importance of compliance were ensured to obtain good control of her RTA. At just over a year, patient had became independently ambulatory.

## Discussion

Renal tubular acidosis is characterized by the kidney’s inability to secrete hydrogen ions or to reabsorb bicarbonate ions resulting in a state of chronic normal anion gap hyperchloremic metabolic acidosis^1,2^. Primarily being a genetic defect, it is also known to be associated with other clinical syndromes such as lupus, hepatitis, vasculitis and other autoimmune diseases^1^. Historically and functionally, RTA has been classified into a group of four distinctive types based on their initial discovery and pathological defects. The Type 1 RTA, which had been diagnosed in our patient, is also known as distal renal tubular acidosis (dRTA) and was the first to be recognized and characterized by distal defect of hydrogen ion secretion with a resultant chronic normal anion gap metabolic acidosis with is accompanied by hypokalaemia. The other varieties of the disease include Type 2 RTA, a proximal tubule defect, Type 3 mixed and Type 4 is secondary to aldosterone deficiency or resistance as well as being the only type known to be associated with hyperkalaemia.

Majority of symptoms arise from the chronic state of metabolic acidosis and when left untreated it poses a multitude of morbidities. Clinical manifestations include hypokalaemia, nephrocalcinosis, hypercalciuria, rickets or osteomalacia^1,2^. Osteomalacia and rickets are classified as metabolic bone diseases, qualitative in nature resulting from inadequate bone mineralization^3^. Rickets occur in bones where growth plates have not achieved maturity as compared to osteomalacia which occurs in the adult age group^3^. However, it must be kept in mind that primary and secondary deficits in vitamin D or impaired metabolism of vitamin D are the most common causation of osteomalacia.

Skeletal complications of RTA classically present as osteomalacia in adulthood or rickets in children. This is a result of the affected kidneys being unable to acidify urine which leads to a chronic state of acidosis which then also affects the vitamin D metabolism^1^ and increases urinary losses of calcium. This directly leads to hypocalcemia and secondary hyperparathyroidism in patients with distal RTA resulting in hypophosphatemia^4^. The resulting secondary hyperparathyroidism contributes to an increased bone resorption rate. Therefore, it is postulated that this combination of hypocalcemia, hypophosphatemia and vitamin D metabolic disturbance results in rickets or osteomalacia in RTA patients^5^. This correlates with the patient’s blood parameters and clinical findings.

Besides that, bone being a critical partaker in buffering excess acid, undergoes accelerated demineralization due to the chronic acidotic environment^2,3^. The eventual sequelae of osteomalacia places patients at risk of developing pathological fractures as in our patient. The pathophysiology differs from that in renal osteodystrophy in which the general glomerular failure leads to a uremic acidemia, hypocalcemic and hyperphostemic blood environment in the events preceding osteoporosis seen in end stage renal failure patients. Besides that, the acidotic state is reversible with proper management, thus suppressing the metabolic abnormalities seen in RTA patients. Therefore, a reversible state allows the patient to reduce or to a lesser extent avoid the risk of osteomalacia, ultimately preventing pathological fractures.

RTA is not new to the medical fraternity. Nevertheless, the awareness of the resulting complications is somewhat lacking. This is crucial as many of these complications can be reduced if the state of chronic metabolic acidosis is addressed. Education and awareness of the disease not only reduces the number of complications, but it goes a long way to ensure the general well-being of patients. The patient in our present report had been diagnosed earlier and had treatment initiated for her RTA. However, she was not compliant with her medications which increased her risk of complications. The patient’s failure to adhere to medical advice led to chronic metabolic acidosis which in turn resulted in accelerated osteomalacia. The eventual low impact trauma resulted in bilateral proximal femoral fractures. Prior to our report, Almeida *et al* were the only ones to have reported bilateral limb fractures in a RTA patient^4^.

In conclusion, metabolic bone resorption in patients with RTA should be closely monitored with prophylaxis being the emphasis to prevent pathological fractures. Good patient education and clinical care will be essential in reducing complications which can be costly to the patient personally as well as the healthcare system. While single bone pathological fractures are common, bilateral limb involvements are rare and consequentially more debilitating.

## Conflict of Interest

The authors declare no conflict of interest.
